# Genetic analysis of four consanguineous multiplex families with inflammatory bowel disease

**DOI:** 10.1093/gastro/goab007

**Published:** 2021-07-13

**Authors:** Noam Ben-Yosef, Matthew Frampton, Elena R Schiff, Saleh Daher, Fadi Abu Baker, Rifaat Safadi, Eran Israeli, Anthony W Segal, Adam P Levine

**Affiliations:** 1 Centre for Molecular Medicine, Division of Medicine, University College London, London, UK; 2 Institute of Gastroenterology and Liver disease, Hadassah Medical Center, Jerusalem, Israel; 3 Institute of Ophthalmology, Moorfields Eye Hospital, University College London, London, UK; 4 Institue of Gastroenterology and Hepatology, Hillel Yaffe Medical Center, Hadera, Israel; 5 Institute of Gastroenterology and Liver disease, E. Wolfson Medical Center, Holon, Israel; 6 Department of Pathology, University College London, London, UK

**Keywords:** inflammatory bowel disease, family study, genetics, homozygosity

## Abstract

**Background:**

Family studies support a genetic predisposition to inflammatory bowel diseases (IBD), but known genetic variants only partially explain the disease heritability. Families with multiple affected individuals potentially harbour rare and high-impact causal variants. Long regions of homozygosity due to recent inbreeding may increase the risk of individuals bearing homozygous loss-of-function variants. This study aimed to identify rare and homozygous genetic variants contributing to IBD.

**Methods:**

Four families with known consanguinity and multiple cases of IBD were recruited. In a family-specific analysis, we utilised homozygosity mapping complemented by whole-exome sequencing.

**Results:**

We detected a single region of homozygosity shared by Crohn's disease cases from a family of Druze ancestry, spanning 2.6 Mb containing the *NOD2* gene. Whole-exome sequencing did not identify any potentially damaging variants within the region, suggesting that non-coding variation may be involved. In addition, affected individuals in the families harboured several rare and potentially damaging homozygous variants in genes with a role in autophagy and innate immunity including *LRRK1*, *WHAMM*, *DENND3*, and *C5*.

**Conclusion:**

This study examined the potential contribution of rare, high-impact homozygous variants in consanguineous families with IBD. While the analysis was not designed to achieve statistical significance, our findings highlight genes or loci that warrant further research. Non-coding variants affecting *NOD2* may be of importance in Druze patients with Crohn's disease.

## Introduction

Inflammatory bowel diseases (IBDs) comprise Crohn's disease (CD) and ulcerative colitis (UC). The pathogenesis of these diseases remains incompletely understood; this may reflect heterogeneity in the underlying causal pathways as a variety of factors may contribute to patients exhibiting similar phenotypes. The heritability of IBD illustrates this complexity. Early studies of families of IBD patients and twin pairs indicated a genetic risk to disease aetiology [1–5]. The discovery of damaging coding variants in the *NOD2* gene associated with CD was the first step in understanding the molecular basis of the disease and also highlighted the role of the innate immune system in CD pathogenesis [6].

Subsequent genome-wide association studies (GWASs) of large cohorts of IBD patients and controls, including those from diverse ethnicities, have identified a growing number of loci and genes associated with IBD, and highlight three notable concepts: (i) the genetic background is complex and polygenic; >200 risk loci have been detected implicating a wide variety of genes (in linkage disequilibrium (LD) with the associated polymorphisms) in CD, UC, or both [7–10]. In fact, more risk loci have been associated with IBD than with any other common complex disease [8]; (ii) most of the associated polymorphisms are common (minor allele frequency (MAF) >5%) and are hence shared by healthy controls (indicating low penetrance) and contribute only modest effect sizes with odds ratios (ORs) typically <1.3 [11]; (iii) even considering the entire spectrum of associated polymorphisms, they account for only 26% (CD) and 19% (UC) of disease variance [12]. This observation, also shared with other complex diseases, is referred to as the ‘missing heritability’.

The heritability gap may be accounted for by several factors including the contribution of environmental exposures (overestimation of the heritability), epistatic effects, epigenetic modifications, or the presence of rare variants with a large effect size [13, 14]. Rare variants are difficult to detect and would demand very large cohorts [15] or the resequencing of candidate genes in population-specific cohorts [16, 17]. Alternatively, a family-designed study complemented by deep sequencing is another approach to detect rare and potentially causal variants. This strategy has been employed in several studies of multiplex families with IBD [18–21] and other comparable complex diseases [22–24].

Inbreeding or recent consanguinity leading to long stretches of homozygosity are known to contribute to Mendelian recessive disease, but may also play a role in complex polygenic diseases [25]. Examples include coronary artery disease [26], schizophrenia [27], Alzheimer's disease [28], and rheumatoid arthritis [29], although evidence is inconclusive. IBD homozygous risk variants have a higher impact than heterozygous variants. This has been shown for *NOD2* [30] and *ATG16L1* [31]. In the case of *NOD2* risk variants, a homozygous variant exceeds the risk simulated by a simple additive effect [30].

Based on the aforementioned concepts, we attempted to identify homozygous and rare variants that potentially contribute to IBD pathogenesis. As such variants are difficult to find in unrelated individuals in outbred populations, we focused on consanguineous families from populations practising endogamy. We report the genetic analysis of four consanguineous families with multiple cases of adult-onset IBD combining genome-wide single-nucleotide polymorphism (SNP) homozygosity mapping (HM) with whole-exome sequencing (WES).

## Methods

### Recruitment

Individuals with IBD and at least one affected first-degree relative were identified from an internal database of patients attending the IBD Unit at Hadassah Medical Center, Israel. Additional patients were referred from other specialist centres in Israel and invited to participate or volunteered following advertisement of the study by the Israel Foundation for Crohn's Disease and Ulcerative Colitis. Further patients were intentionally sought from clinics that treat large numbers of individuals from the Arab Muslim (AM) and Druze populations, as these populations have consanguinity rates that exceed 40% [32]. Interviews were initially conducted by telephone with subsequent visits to the family if there were confirmed affected first-degree relatives.

The study protocol was approved by the Institutional Review Board of Hadassah Medical Center (0557–13) and the Israeli Ministry of Health (042–2015). Consenting participant identities were coded at sample collection and the participant identity-code match was known only to the recruiting physician.

Family members were questioned regarding family background, consanguineous relations, and the presence of IBD or other immune-mediated diseases in the extended family. Diagnosis and phenotypic details were ascertained by review of medical files. The absence of disease in unaffected family members was based on specific questioning with an emphasis on chronic gastrointestinal symptoms. Saliva samples were obtained and DNA isolated according to standard procedures [33].

### Genetic-analysis overview

Four families with affected offspring from consanguineous matings were identified from the recruited cohort that comprised a total of 60 multiplex families. We performed SNP microarray genotyping and subsequent HM, complemented by WES. For downstream variant prioritisation, a series of filters were applied based on population frequency, *in silico* prediction of pathogenicity, gene function and expression, and previous association with IBD.

This study focused on private and rare variants within families and was not powered or designed to achieve statistical significance. The analysis was family-specific, as the diverse background of the families did not suggest the existence of a shared risk variant.

### Family pedigrees

Pedigrees were drawn with HaploPainter [34] and modified to protect the anonymity of participants without altering the distribution of individuals and sex of cases.

### Genotyping and quality control

All individuals recruited from the four consanguineous families (*n *=* *38) were genotyped using the Illumina Infinium HumanCoreExome-24 Bead Chip (Illumina, San Diego, CA) and processed with the Illumina GenomeStudio software. Quality control was undertaken using PLINK: samples were removed if missing >5% of SNPs and then SNPs were removed if they were missing in >5% of samples or deviated significantly from Hardy–Weinberg Equilibrium (Fisher’s exact test *P*-value <5* × *10^–8^) [35].

The sex of each sample was verified by the homozygosity rate of common variants (MAF >0.05) on the X chromosome (excluding the pseudoautosomal regions). Pedigree structure and relatedness between samples were verified by kinship coefficient calculations using KING [36].

### Homozygosity mapping

Regions of homozygosity (ROH) were detected using HomozygosityMapper [37]. Common (MAF >0.05 for the combined gnomAD (https://gnomad.broadinstitute.org/) population) SNPs were used as input. Default software parameters were employed enabling the ‘require genetic homogeneity’ feature, thus limiting the detection of ROH to those shared by all cases and of identical genotype. Analyses were performed with and without the exclusion of long ROH in unaffected family controls, allowing for the possibility of incomplete penetrance.

A minimum threshold for ROH length of 1 Mb was defined; this represents ROH caused by recent consanguinity as implied by the pedigree (expected to contain several mega bases), as well as homozygous segments resulting from a more distant history of inbreeding, as suggested by the reported history of the families [38]. A minimum threshold of 50 continuous identical SNPs was required to exclude regions identical by state rather than identical by descent and large regions with low SNP density (Supplementary Methods).

Protein-coding genes within the ROH coordinates (human genome build GRCh37) were identified using the Ensembl BioMart tool [39]. The presence of IBD GWAS loci within ROH was examined using the top SNP reported in the loci [11].

HM analysis was complemented with manual inspection of all rare variants identified through WES (described below) that were located within each ROH. The WES data were also used to confirm the identification of ROH, with the absence of heterozygous variants within these regions.

### Whole-exome sequencing

Exome capture and library preparation were performed using the BGI 59 Exome Enrichment Kit (BGI, China). One sample failed library preparation quality control. Paired-end sequence reads (150 bp) were generated using the Illumina HiSeq 2000 system (Illumina, San Diego, USA).

#### Read alignment and variant calling

Sequence reads were aligned to the human reference genome hg19 using Novoalign (version 3.02.08). Samples were processed jointly with 5,285 samples comprising the UCL Exome Sequence Consortium, a local collection of exomes from a variety of cohorts. Variants were called using the Genome Analysis Tool Kit (GATK) [40] according to best practices [41] with local realignment around InDels, followed by joint variant calling and variant quality score recalibration (VQSR).

Several quality-control filters were applied to the variants. First, we required a VQSR truth tranche ≤99.5% for SNPs and ≤99.0% for InDels. Next, we applied filters to individual genotypes and changed those failing to ‘no-calls’: Genotype Quality should be ≥30, for heterozygous calls the read depth of the alternative allele should not diverge significantly from 50:50 (chi-squared test *P *<* *0.001), and for heterozygous or alternate homozygous calls the read depth of the alternate allele should be ≥3. Finally, we only retained variants with a no-call rate <0.25.

#### Variant annotation, filtering, and prioritization

Variants were interpreted and annotated using the Ensembl Variant Effect Predictor tool (VEP) [42] for the predicted impact on Ensembl gene transcripts and population frequencies in gnomAD [43]. Missense variants were annotated with their predicted effect on protein function *in silico* using PolyPhen-2 [44], SIFT [45] (with VEP), and CADD score [46]. The Druze and Arab Middle-Eastern populations are not represented in gnomAD; variant frequencies from the Greater Middle East Variome Project (GMEV) [47] were thus included. The GMEV consists of WES-derived variants from >1,000 unrelated subjects of Middle-Eastern descent, enabling variants common amongst such populations to be identified and excluded [47] (Supplementary Methods).

Variants were defined as ‘damaging’ if they were either a frameshift, start/stop altering, splice acceptor/donor variants, or missense variants predicted to be deleterious by either PolyPhen-2, SIFT, or CADD (CADD score >20). Three classes of population allele frequency were defined: ‘uncommon’ as 1%* *<* *MAF* *<* *5%; ‘rare’ as 0.1%* *<* *MAF* *<* *1%; and ‘very rare’ as MAF* *<0.1%. For the Druze and AM families, these thresholds had to be satisfied in both the gnomAD combined population and the GMEV. Similarly, for the Ashkenazi Jewish (AJ) family, these thresholds had to be satisfied in both in the gnomAD combined and AJ-specific populations.

To maximize the sensitivity to detect homozygous variants of potential interest, we utilised a non-stringent threshold for predicted deleteriousness (retaining variants predicted to be deleterious by any predictive model) or population frequency (retaining uncommon variants). We note that, under Hardy–Weinberg Equilibrium, even uncommon variants should be exceptionally scarce in the homozygous state.

Following annotation and filtering, homozygous ‘damaging’ and ‘uncommon’ (or of lower frequency) variants present in the proband of each family were identified. Each of these variants was further examined separately considering the segregation within cases in the family and absence in unaffected family controls. Furthermore, the encoded protein's function (in UniProt [48]), expression profile (as per BioGPS [49]), and prior association with IBD (described below) were examined. Finally, we queried the IBD Exomes Browser [50] for each specific variant to determine whether the gene is enriched for variants affecting IBD risk.

### IBD-associated genes

To focus our search for candidate variants, a set of genes with known IBD association was defined. This included 251 genes reported by Mirkov *et al.* [11] from loci associated with IBD by GWAS, 67 genes reported in cases of monogenic phenotypes of very-early-onset IBD [51], and additional genes from a literature search for association studies or direct-sequencing studies. After removing duplicates, a list of 366 IBD-associated genes was generated (Supplementary Table 1A). Our aim was to achieve a permissive and comprehensive list, which we refer to as the ‘extensive’ IBD-association list.

A second list of 20 ‘top genes’ that have been confidently implicated in IBD risk by fine-mapping or with functional data [15] was also generated (Supplementary Table 1B). The enrichment of variants within genes from the ‘extensive’ gene list and the ‘top genes’ list was examined. This strategy was separately employed for all variants and for the homozygous variants. To avoid bias due to pedigree structure, the number of homozygous variants was only compared between siblings, as they are expected to share an equal fraction of their genome in the homozygous state. Finally, cases and family controls were screened for a set of coding functional variants directly implicated in IBD pathogenesis [52–54] (Supplementary Table 1C).

## Results

### Description of families

**Figure 1.**
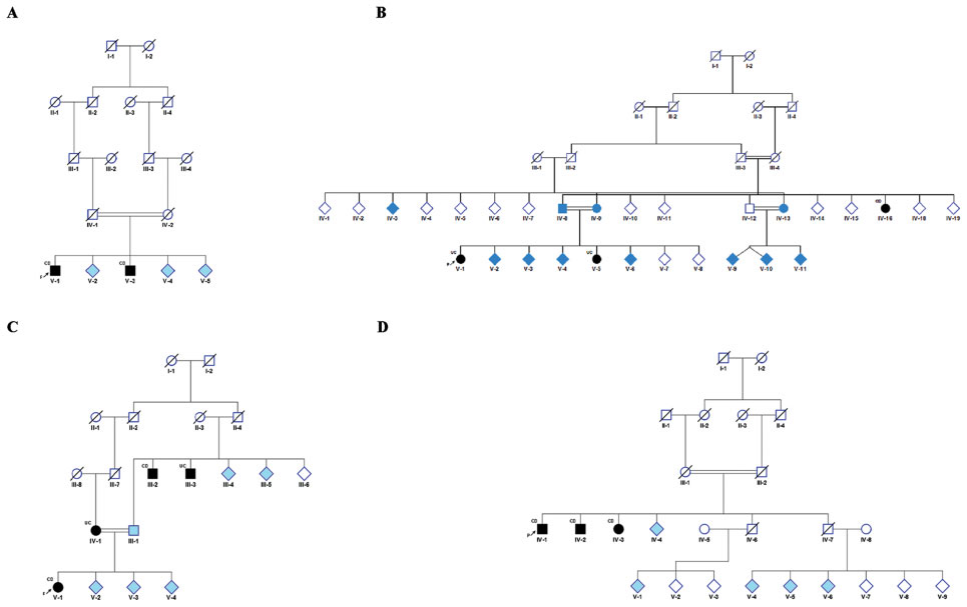
Pedigrees of the four consanguineous families with inflammatory bowel disease (IBD). (A) Arab Muslim family with Crohn’s disease (CD) as the major phenotype. (B) Arab Muslim family with ulcerative colitis (UC) as the major phenotype. (C) Druze family. (D) Ashkenazi Jewish family. IBD cases are coloured in black. Unaffected family controls who were also exome sequenced are coloured in light blue.

A total of 38 individuals from four consanguineous families participated in the study. This comprised 8 CD cases, 4 UC cases, and 26 unaffected family members. Pedigrees for the families are provided in Figure 1. The case with the most severe phenotype at the time of study recruitment was designated as the proband in each family. Two families of AM ancestry expressed either CD or UC as the major phenotype. The other two families were of Druze and AJ descent. For purposes of clarity and ease of reading, the families are designated as AM-CD, AM-UC, DR, and AJ, respectively.

Families AM-CD and AM-UC are each members of a different tribe (*chamulla*) tracing their ancestry back 200–300 years. Today, each tribe numbers a few thousand members and marriage is generally, although not exclusively, within the tribe. DR have a unique social and demographic structure, with customs that strongly favour marriage within the village, and mating outside the religion is forbidden [55]. AJ are a genetically distinct population, estimated to have arisen from ∼350 individuals 25–32 generations ago [56].

In family AJ (Figure 1D), two siblings died from ischemic heart disease diagnosed when >60 years of age and had no prior history of gastrointestinal diagnoses or symptoms. Four descendants of these individuals were available for sequencing; if any descendant was homozygous for the reference allele, then homozygosity of the parent for the candidate variant (alternate allele) was inferred to not be possible.

The average age of disease onset amongst the cohort was 42.9 years for CD and 26.5 years for UC, but varied substantially between families, with the AJ family having a relatively later age of onset (average 56.3 years). Additional clinical information is provided in **Table 1**.

**Table 1.** Clinical information for cases with inflammatory bowel disease

Family	Patient ID	Relation to proband	Gender	Diagnosis	Age at diagnosis	Location	Behaviour (CD)
AJ	IV-1	Proband	Male	CD	59	Ileal	Stricturing and penetrating
	IV-2	Sibling	Male	CD	51	Ileal	Stricturing
	IV-3	Sibling	Female	CD	59	Ileal	Non-stricturing, non-penetrating
DR	V-1	Proband	Female	CD	22	Ileocolonic with upper gastrointestinal involvement	Non-stricturing, non-penetrating
	III-2	Uncle	Male	CD	45	Ileocolonic	Non-stricturing, non-penetrating
	IV-1	Mother	Female	UC	21	Left-sided/proctitis	–
	III-3	Uncle	Male	UC	42	Left-sided/proctitis	–
AM-CD	V-1	Proband	Male	CD	32	Ileocolonic	Stricturing
	V-3	Sibling	Male	CD	33	Ileal	Non-stricturing, non-penetrating
AM-UC	V-1	Proband	Female	UC	28	Left-sided	–
	V-5	Sibling	Female	UC	15	Proctitis	–
	IV-16	Aunt	Female	CD	42	Ileal	Non-stricturing, non-penetrating

AJ, Ashkenazi Jewish family; DR, Druze family; AM, Arab Muslim family; CD, Crohn’s disease; UC, ulcerative colitis.

Patient IDs refer to the pedigrees in Figure 1.

### Data generation

#### Genome-wide SNP genotyping

Members of the four consanguineous families were genotyped with a SNP microarray (*n *=* *38). Two individuals failed to pass genotyping quality-control criteria (missing genotypes >5%); both were cases from family AM-CD and therefore this family was not included in the HM analysis. Following quality-control filtering and removal of SNPs with duplicate IDs, 251,716 markers across the autosomes were available for HM, covering 2,790 Mb of the autosome (Supplementary Methods).

#### Whole-exome sequencing

WES analysis was performed on 37 individuals from the four families following successful library preparation. Following quality control, 95.4% of the exome was covered at 5× and 87.6% at 20×, with an average read depth of 89.1×. A total of 32,272 variants were common to the WES and SNP-microarray genotyping for which the call concordance rate was >0.99. The average number of exome-wide (coding and splice site only) sequence variants in the autosomes per individual was 19,550.

### Homozygosity mapping

ROH shared by affected family members with concordant IBD subtypes, following manual inspection of the region and confirmation of identity by descent, are presented in **Table 2**. No ROH were shared among the UC cases from family DR.

**Table 2.** Regions of homozygosity (ROH) detected by homozygosity mapping

Family	Chr	Start^a^	End^a^	Length (Mb)	Number of SNPs	Overlapping ROH in family controls	Presence of IBD GWAS locus^b^	Known IBD-associated genes located in region	Number of protein- coding genes^c^	Number of uncommon variants^d^	Rare variants predicted to be damaging^d^
AM-UC	1	37,013,031	46,037,357	9.0	772	V-3	No IBD GWAS loci	None	123	7	0
	8	137,523,046	142,950,775	5.4	612	V-3	No IBD GWAS loci	None	14	2	*DENND3*
	9	124,704,304	141,022,295	16.3	1,539	V-2, V-9, V-10	rs10781499, OR 1.19 (IBD), *P*-value 4.38E-56, RAF 0.5	*CARD9, SDCCAG3, PMPCA*	281	18	*CERCAM, WDR38*
	15	54,343,188	79,365,780	25.0	2,154	None	rs17293632, OR 1.11 (IBD), *P*-value 2.71E-20, RAF 0.14	*SMAD3*	220	16	*AAGAB*
	15	51,663,597	52,878,719	1.2	57	IV-9, IV-13, V-2	No IBD GWAS loci	None	14	3	0
DR: CD cases	2	220,753,783	222,044,974	1.3	141	III-1, IV-1, V-2, V-3, V-4	No IBD GWAS loci	None	0	0	0
	16	48,846,170	51,468,005	2.6	359	None	rs2066844, OR 2.003 (IBD), *P*-value 2.27E-217, RAF 0.06	*HEATR3, ADCY7, BRD7, NOD2*	13	0	0
AJ	2	183,467,456	190,452,586	7.0	451	IV-4	rs144344067, OR 1.12 (IBD), *P*-value 1.29E-08, RAF 0.89	*ITGAV, FAM171B*	21	5	0
	4	53,383,129	54,396,077	1.0	55	None	No IBD GWAS loci	None	6	0	0
	12	22,890,730	26,139,522	3.2	361	None	No IBD GWAS loci	None	11	0	0

AM, Arab Muslim family; DR, Druze family; AJ, Ashkenazi Jewish family; CD, Crohn’s disease; UC, ulcerative colitis; Chr, chromosome; SNP, single-nucleotide polymorphism; IBD, inflammatory bowel disease; GWAS, genome-wide association study; OR, odds ratio; RAF, risk-allele frequency.

ROH shared between cases in each family. Minimum threshold for ROH identification is length >1 Mb and ≥50 SNPs.

^a^ROH positions refer to GRCh37.

^b^Evaluated by the position of the SNP with the smallest *P*-value in an IBD GWAS locus. SNP rsID, OR, *P*-value for association, and RAF in the European population are provided, adapted from Ref. [11] and originally reported in Refs [8–10, 52].

^c^Protein-coding genes within the region were identified using the BioMart tool available from Ensembl. A complete list of genes in each ROH is available in Supplementary Table 2.

^d^Minor-allele frequency <0.05 for uncommon variants and <0.01 for rare variants.

Together, these ROH contain 703 protein-coding genes (∼3.6% of all protein-coding genes covered by HomozygosityMapper; a complete list of these genes is provided in Supplementary Table 2). They are significantly enriched in genes from the top 20 IBD-associated genes (‘top genes’ list [15], 3/20 genes; *CARD9*, *SMAD3*, and *NOD2*; OR 4.7, *P *=* *0.03), but not with broader sets of genes previously associated with IBD (10/366 IBD-associated genes from our ‘extensive’ list and 4/242 GWAS loci [11] are contained in these ROH). Only four rare variants predicted to be damaging were located within these ROH, all in family AM-UC (**Table 2**).

The two affected siblings from family AM-UC shared four ROH spanning a total of 55.8 Mb (2% of the covered genome). This was 10-fold longer than the total shared ROH in other families—an expected finding given the pedigree structure in family DR and the requirement for overlapping ROH for three cases in family AJ. Combining HM in family AM-UC with the affected aunt (diagnosed with CD, Figure 1B) did not identify any ROH passing our threshold of 1 Mb (the longest shared region is 0.87 Mb long containing 51 identical homozygous SNPs and all other stretches are shorter than 0.5 Mb). Similarly, in family DR, combining HM for all four affected members with any IBD subtype identified only one ROH longer than 1 Mb located on chromosome 11 near the centromere, which was also shared with four unaffected members of the family. A manual inspection of this region reveals that it is not a true ROH; it encompassed only 49 continuous SNPs and includes 2 heterozygous SNP calls that were incorrectly considered by the software to be erroneous genotypes as they were flanked on both sides by a series of identical homozygous SNPs. This illustrates the complexity of SNP-microarray-based homozygosity-mapping analysis in highly inbred families, favouring a stringent threshold of continuous identical SNPs to define true autozygosity, especially in genomic regions with low SNP-marker density.

**Figure 2.**
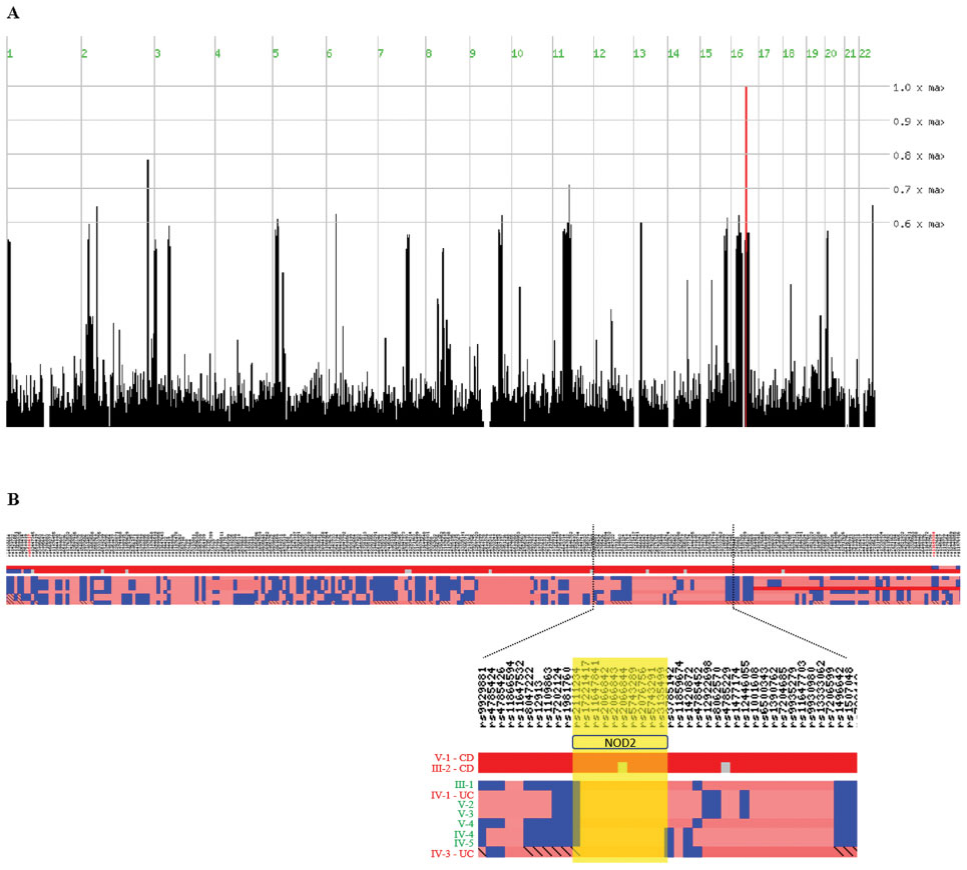
**Region of homozygosity on chromosome 16 in the Druze family.** (A) Whole-genome view of the region of homozygosity (ROH) shared by Crohn's disease cases (V-1 and III-2) in the Druze family. The height of the bars corresponds to the number of continuous homozygous markers shared by both cases and the width corresponds to the length of the ROH. The region with the highest score (red bar) is located on chromosome 16 and contains 359 single-nucleotide polymorphism (SNP) markers, spanning 2.6 Mb and surrounding *NOD2*. (B) Genotype view for region of homozygosity on chromosome 16. Each column represents a SNP marker and each row an individual in the family; the two CD cases are at the top. The blue colour represents a heterozygous call for the marker and red represents the homozygous calls (darker shades of red reflect the number of continuous homozygous calls; the grey colour indicates a ‘no-call’ for that marker). The position of *NOD2* is indicated by the yellow box. Examination of the exome sequence confirms that the two CD cases are indeed homozygous across and surrounding *NOD2*, but other members of the family are heterozygous.

The two CD cases from family DR (V-1 and III-2) shared a single ROH located on chromosome 16 (Figure 2A) spanning 2.6 Mb containing the *NOD2* gene. Overlapping ROH were not observed for any other member of the family (Figure 2B). This ROH also contains *BRD7* and *HEATR3*, both associated with CD (either independently or due to their linkage disequeilibrium (LD) with *NOD2*) [57]. Interestingly, the three well-established CD associated *NOD2* risk variants (fs1007insC, R702W, and G908R) were homozygous wild type in these CD cases. In fact, the entire region contained only four intronic variants and four coding synonymous variants, all common (MAF >0.05). Two of these are synonymous variants in *NOD2* (S178 and R587, in LD) that are significantly associated with CD *protection* (OR* *0.77, *P *<* *1 × 10^–16^ in the IBD Exome Browser). The robustness of this finding was verified by repeating the analysis with variable parameters, implicating only one additional ROH on chromosome 2, which is also shared by five other members of the family and contains no protein-coding genes.

Individuals in family DR were found to have a greater fraction of their genome within ROH than expected based on the pedigree alone, suggestive of historical inbreeding. Indeed, long ROH were also detected in individual III-2 and his siblings (generation III in Figure 1C, Supplementary Methods). Therefore, to evaluate the probability that a specific region on chromosome 16 containing *NOD2* would be identically homozygous between the CD cases in this family, a hypothetical pedigree was constructed in which individual III-2 is the offspring of a first-cousin mating. Meiotic events including recombination were simulated by gene dropping for the entire autosome in the pedigree 10,000 times (Supplementary Methods). The probability that both affected individuals of relevance were identically homozygous for a specific 1 cM region on chromosome 16 (at the approximate position of *NOD2*) was estimated as *P *=* *0.0021.

### Exome-wide variant analysis

Focusing on the homozygous variants, each individual had an average of 6,749 for any (regardless of allele frequency), 39.7 for uncommon (MAF <0.05), 11.8 for rare (MAF <0.01), and 3.8 for rare and damaging variants. Examining only offspring of observed consanguineous matings, the corresponding averages were higher, at 60.0, 19.6, and 7.0, respectively. An overview of the homozygous variants observed in these families is provided in Supplementary Table 3.

No CD case carried any of the known *NOD2* risk variants. All three CD cases in family AJ were homozygous for the common T300A risk allele (OR* *1.2^8^) in *ATG16L1*, as were five unaffected controls in this family. No other case was homozygous for any of the functional variants directly implicated in IBD pathogenesis [52–54]. The distribution of potentially damaging homozygous variants in IBD-associated genes is outlined in Supplementary Table 4.

The probands in each family had 9–18 (average 13.5) homozygous, uncommon, and damaging variants. A description of these 54 variants is provided in Supplementary Table 5. For further examination, variants were prioritised if they were homozygous in both affected siblings in family AM-CD or family AM-UC or at least two of three affected siblings in family AJ. In family DR, the complex pedigree structure and case relatedness did not favour a prioritisation strategy so all variants were initially examined, unless they were also common in unaffected family controls. We further excluded variants in unknown genes or genes that have a specific function or expression profile that does not suggest involvement in IBD pathogenesis, leaving 19 variants. The prioritized variants are described in **Table 3** and with additional detail in Supplementary Table 6.

**Table 3.** Prioritized homozygous variants

Family	Chr	rsID	Gene	Consequence	Amino-acid change	SIFT	PolyPhen	CADD score	gnomAD MAF	Population- specific MAF^a^	Homozygous in controls^b^
AM-CD	10	rs56226109	*FGFR2*	Missense	S57L	Tolerated	Benign	22.4	0.0049	0.0065	0/3
	10	rs143892520	*DMBT1*	Missense	D560E	Deleterious	Unknown	12.66	0.015	–	0/3
AM-UC	8	–	*DENND3*	Missense	R487S	Deleterious	Possibly damaging	18.83	–	–	1/10
	9	rs5794	*PTGS1*	Missense	V481I	Deleterious	Benign	23.3	0.0072	0.0146	3/10
	9	–	*WDR38*	Missense	R51W	Deleterious	Possibly damaging	28.4	–	–	3/10
	9	rs146651928	*CERCAM*	Missense	Q187E	Deleterious	Probably damaging	22.9	0.0006	0.0005	3/10
	15	–	*AAGAB*	Missense	D312N	Tolerated	Possibly damaging	25.8	–	–	0/10
DR	2	rs148167737	*MAP4K3*	Missense	A410T	Tolerated	Benign	23.9	0.0008	–	1/6
	2	rs377671536	*MAP4K4*	Missense	Q515E	Tolerated (LC)	Possibly damaging	23.3	0.0002	0.0005	0/6
	9	rs3739740	*NIPSNAP3B*	Missense	K154E	Deleterious	Possibly damaging	27.7	0.0267	0.0347	1/6
	15	rs185529473	*WHAMM*	Missense	R725W	Deleterious	Possibly damaging	29.5	4.47E-05	–	0/6
	15	rs61752778	*ADAMTSL3*	Missense	P821S	Tolerated	Probably damaging	24.4	0.0163	0.0091	0/6
	15	rs61731243	*AKAP13*	Missense	E1106G	Deleterious (LC)	Benign	16.55	0.0415	0.0267	0/6
	15	rs55798315	*LRRK1*	Missense	P543S	Deleterious	Probably damaging	29.8	0.0044	0.0171	1/6
AJ	4	rs112033303	*COQ2*	Stop gained	R22X	–	–	23	0.0166	0.0304	0–1/3
	2	rs138440701	*IL36B*	Missense	I110T	Deleterious (LC)	Benign	3.083	0.013	0.0222	0–1/3
	9	rs34552775	*C5*	Missense	L354M	Deleterious	Possibly damaging	25	0.0054	0.0044	0–1/3
	19	rs75841596	*PALM3*	Missense	D604N	Deleterious	Possibly damaging	26.8	0.0245	0.0319	0/3
	19	rs75251420	*MYO9B*	Missense	V1700M	Deleterious	Benign	23.9	0.0014	0.0024	0/3

AM, Arab Muslim family; DR, Druze family; AJ, Ashkenazi Jewish family; CD, Crohn’s disease; UC, ulcerative colitis; Chr, chromosome; LC, low confidence; gnomAD, Genome Aggregation Database; MAF, minor allele frequency (missing MAF: non-existent in data set).

Family-specific variants identified by whole-exome sequencing. Variants were prioritised if they satisfied all of the following: homozygous, predicted to be damaging, allele frequency <0.05, segregated in the affected cases, and could not be excluded based on an unrelated specific function or gene expression.

^a^Using MAF in the Greater Middle East Variome (for families AM-CD, AM-UC, and DR) or the gnomAD Ashkenazi Jewish population (for family AJ).

^b^In the AJ family, estimation for homozygosity for the deceased unaffected siblings was based on whole-exome sequence data from their descendants.

In family AM-CD, all four prioritised variants were present in a 12-Mb region on chromosome 10. A manual inspection of all variants in this region confirmed that it is part of a long ROH shared by the two affected brothers, but not their unaffected siblings. In families DR, AM-CD, and AM-UC, incorporating the GMEV as a reference for allele frequency altered the MAF class in 4/23 variants (3 changed from ‘rare’ to ‘uncommon’, 1 from ‘very rare to rare’).

As expected, all variants identified on the basis of their presence within a ROH identified by HM were independently detected by WES filtering and prioritisation. In contrast, three homozygous variants derived from the WES and shared by all cases (one in family AJ and two in family AM-UC) were not identified with HM. One was located on the X chromosome and would not be detected, as only the autosome was analysed by HM. The other two were located in very short ROHs, far below our threshold for identification. In family AJ, 9/10 variants shared by two affected siblings would have been detected, with HM targeting two cases separately instead of all three together.

## Discussion

Research efforts aiming to elucidate the genetic susceptibility to IBD have focused on common variants with modest effect size derived from large-scale GWASs, alongside attempts to unravel rare and highly penetrant variants in population-specific cohorts or family-focused studies. Familial cases have the potential to harbour rare causal variants that would be difficult to identify in cohorts of unrelated outbred cases and controls [13]. High-impact variants are likely to induce substantially increased risk when both alleles are pathogenic. In CD, this is exemplified by the *NOD2* frameshift variant, for which a homozygous variant is associated with greater disease risk [30], earlier onset [58], and more severe phenotype [59].

In order to target potentially causal and rare homozygous variants, we recruited multiplex families from selected homogenous populations. Despite focusing on populations with high consanguinity rates [32] in the AM and Druze, we only identified three multiplex consanguineous families with IBD. This likely reflects the lower background disease prevalence in the AM and Druze in Israel [60, 61].

Our analysis strategy combined HM and WES. The most intriguing finding derived from HM is a 2.6-Mb ROH shared by both CD cases in family DR, but not any other member of the family. This region harbours 13 protein-coding genes, including *NOD2*. Interestingly, the entire region included only four coding variants, all of which are common synonymous variants, ultimately leading to our impression that this ROH may contain a non-coding sequence variant that alters *NOD2* function or expression. The presence of known *NOD2* risk variants is significantly lower in the non-Jewish than in the Jewish CD cases in Israel, raising the possibility that novel *NOD2* variants determine disease susceptibility in the non-Jewish population [62]. We suggest that further study of this locus, including examination for non-coding variants, particularly amongst the Druze population, may be warranted. Apart from the abovementioned ROH, we did not detect an enrichment of IBD-associated genes within ROH shared by cases using our extensive list of 366 genes.

WES identified a total of 54 uncommon and damaging variants that were homozygous in the probands. Only four of these variants are located in genes previously implicated to have a role in IBD (*DMBT1*, *LRRK1*, *MUC1*, and *MYO9B*). Additional prioritisation steps narrowed the list to 19 variants (2–7 per family) that potentially contribute to disease pathogenesis in a family-specific manner. While this study was not designed to achieve statistical significance or experimental validation for their role in IBD aetiology, we suggest that several variants residing in genes with a role in autophagy or innate immunity warrant additional consideration in future studies:

- ***LRRK1*** is essential for autophagosome and lysosome fusion through its regulation of the activation-deactivation cycle of Rab7 [63]. A locus containing the *LRRK1* gene has been associated with CD [64]. Missense variants in *LRRK2*, a close paralogue, have been causally implicated in CD [54].
- ***WHAMM*** is an actin nucleation-promoting factor with a key role in autophagosome formation. *WHAMM* directs the activity of the Arp2/3 complex for autophagosome biogenesis through an actin-comet tail motility mechanism, and *WHAMM* expression correlates with the number and size of autophagosomes [65, 66].
- ***DENND3*** activates Rab12, which facilitates the trafficking of autophagosomes towards the lysosomes [67]. The *p*. *Arg487Ser* variant does not exist in any population in gnomAD or GMEV, and is located within the ‘linker region’ required to link *DENND3* to Rab12.
- ***C5*** is a terminal complement factor common to all complement pathways. C5 is cleaved to C5a, a potent chemoattractant and anaphylatoxin, and C5b, which initiates the formation of and contributes to the membrane attack complex [68]. The candidate variant *p*. *Leu354Met* is located within C5b. Rare variants in the C5b chain are associated with recurrent bacterial infections [68]. Earlier studies suggest association of CD with impaired complement function [69]. Reduced immunity and clearance of bacterial intrusions may lead to the chronic bowel inflammation in CD [70, 71].

Two unresolved questions regarding the genetic basis of familial IBD and other complex polygenic diseases with late onset underlie the intention and design of this study. (i) Is the increased familial risk a result of private high-impact variants or the accumulation of many common low-effect polymorphisms? (selected arguments reviewed in Ref. [72]). (ii) Does increased homozygosity across the genome contribute to disease risk? Studies of complex polygenic diseases have provided both positive [26, 27, 73, 74] and negative [75, 76] results. Even if homozygosity increases disease risk, it may not necessarily reflect the presence of rare loss-of-function variants residing on both alleles, which was the focus of our study, but could derive from the combined effects of many common risk alleles. To our knowledge, only one study has attempted to determine the contribution of consanguinity to IBD risk and it did not reach definitive conclusions; however, no genetic data were available and pedigree information was incomplete [77].

One methodological issue arising from this study regards the added benefit of combining HM with WES, as all homozygous variants should be detected with WES alone. HM can implicate regions of interest if they harbour genes associated with the studied phenotype and allow careful reconsideration of variants that were filtered by the WES analysis pipeline (e.g. if they were not predicted to be damaging according to the study definition). In addition, WES filtering for uncommon variants would not have detected our most interesting finding from HM—the ROH on chromosome 16 in family DR.

We note two important limitations of this study. First, we did not replicate the identified variants in an unrelated case–control cohort. However, this would be challenging given the rarity of the variants prioritised. Additionally, validation would require using similar populations, and we are unaware of the presence of IBD case–control cohorts with exome data from the Druze and AM populations. Second, exome-focused analysis covers only a small fraction of the genome. We have chosen this strategy not only because of reduced costs and feasibility for systematic bioinformatic annotation, but mainly because high-impact penetrant variants are more likely to reside within the protein-coding sequence [52].

In summary, we have performed a comprehensive genetic analysis of four multiplex and consanguineous families in an attempt to identify rare, high-impact, homozygous IBD-associated variants. We have identified a ROH spanning 2.6 Mb surrounding the *NOD2* gene and shared by two CD cases, lacking potentially damaging coding variants. This region should be explored in the future in studies targeting the unique Druze population. We have suggested four genes, namely *LRRK1*, *WHAMM*, *DENND3*, and *C5*, which are associated with autophagy and/or innate immunity and contain rare and damaging variants that may be aetiologically involved with IBD in these families and also warrant further research.

## Supplementary Data

Supplementary data is available at *Gastroenterology Report* online.

## Authors’ contributions

N.B.Y.: study design, participant recruitment, data collection and interpretation, data analysis, drafting of the manuscript; M.F. and N.P.: data analysis; A.P.L. and E.R.S.: participant recruitment, data analysis; S.D., F.A.B, and F.S.: participant recruitment; E.I.: participant recruitment, interpretation of data; A.W.S.: study design and supervision. All authors critically revised the manuscript and approved the final version to be submitted.

## Funding

This work was supported by the Charles Wolfson Charitable Trust and the Medical Research Council.

## Supplementary Material

goab007_Supplementary_Data
