# Supplementary material for: Genetic analysis of four consanguineous multiplex families with inflammatory bowel disease
Source: Gastroenterol Rep (Oxf). 2021 Jul 13;9(6):521–32. doi: 10.1093/gastro/goab007 (PMC8677555; doi:10.1093/gastro/goab007)
Supplement: goab007_Supplementary_Data [file goab007_supplementary_data.zip › Supplementary_Methods.docx]

**Supplementary Methods**

**Homozygosity mapping**

For homozygosity mapping (HM), only common (minor allele frequency (MAF) > 0.05 in non-Finnish European population) SNP markers were used. Running the analysis with low frequency SNPs inflates the results with long stretches of homozygosity derived from the presence of consecutive homozygous SNPs of the common allele. **Table S1** details the number of SNPs available for analysis following genotyping quality control.

**Table S1**. SNP markers available for homozygosity mapping

|  | Number of SNP markers |
| --- | --- |
| Start: Genome wide SNPs | 549,167 |
| Marker-level quality control | 544,149 |
| SNPs with MAF > 0.05 for homozygosity mapping | 263,657 |
| SNPs accepted by Homozygosity Mapper (Duplicates removed, Insertion/deletion markers removed) | 263,455 |
| SNPs available for homozygosity mapping (only autosomes included) | 251,716 |

***HomozygosityMapper***

Regions of homozygosity (ROH) were detected with HomozygosityMapper [1] (<http://www.homozygositymapper.org>) using the microarray derived SNP genotypes as input. HomozygosityMapper is a web-based tool designed to detect runs of homozygosity. It analyses the data independently of the reference allele frequencies or pedigree structure. ROH are detected as long stretches of homozygous SNPs. Each region receives a score equal to the number of continuous homozygous SNPs within it. When two or more cases have overlapping ROH, the score for the overlapping segment equals the sum of the scores of the ROH from each case. To detect ROH of interest, the software identifies the ROH with the highest score, and then highlights all ROH that achieved the maximum score or close to it.

The user can amend important parameters that will affect the final result:

1. Threshold for identifying ROH. The default is set to 0.8 x Max score but reducing this threshold will achieve more ROH for consideration.
2. Maximum block length. When this parameter is not limited, the final score may be inflated by one individual with an exceptionally long ROH. For example, in our cohort of consanguineous families, some individuals had ROH of more than 5,000 SNPs. In such a case as an example, only overlapping ROHs from cases within this region will be identified, but it will be difficult to distinguish between very short and non-informative segments of even singular SNPs and longer segments of over a hundred SNPs, as they will all achieve a score near 5,000. The recommended default parameter reflects the density of the SNP marker, and in the case of ~250K markers, the maximum block length is set to 250. However, reducing the maximum block length will identify a growing number of ROHs that pass the threshold score.

First, the default software parameters were used, enabling the "require genetic homogeneity" feature to limit the detection of ROH to those shared by all cases and of the same genotype. We ran the analysis both with and without excluding long ROH in unaffected controls in the family, allowing for the possibility of reduced penetrance. We defined a minimum threshold for ROH length of 1 Mb, representing ROH caused by recent consanguinity as implied by the pedigree (expected to contain several mega base pairs [2]), as well as homozygous segments resulting from a more distant history of inbreeding [2], as suggested by the reported history of the families. In addition, a continuous series of homozygous SNPs, may actually represent regions that are identical by *state*, and not truly identical by descent. In other words, these regions reflect the common SNPs in a given population group, but do not necessarily harbor identical sequences within them. To limit the identification of ROH to those more likely to be identical by descent we defined a minimum threshold of 50 continuous identical SNPs.

To confirm that no ROH of potential interest were being filtered out, HM was run again with a reduced limitation of the maximum block length, so that it would be sensitive enough to detect shorter ROH. If additional ROH were detected that passed the threshold of 1 Mb length and 50 identical SNPs, they were added to the final results. We continuously reduced the threshold for ROH detection and the maximum block length, until no additional ROH were identified.

Additional pitfalls required attention. Firstly, HomozygosityMapper allows for single heterozygous calls within long ROH and considers them to be false negatives. However, in the case of individuals from inbred populations, single heterozygous calls surrounded by identical homozygous SNPs are more likely to be true calls, since the neighboring SNPs represent the common allele in this population. Secondly, in regions where SNP marker density is sparse, continuous homozygous SNPs are less specific to detect true regions that are identical by descent. To overcome these difficulties, each region identified was examined manually for the presence of heterozygous SNP calls (counted as false negative), for the proximity to the centromere (due to low marker density) and for the presence of heterozygous variants identified by whole exome sequencing. If any of these suggested the region is not identical by descent, then this ROH was excluded.

***Estimation of inbreeding coefficient***

The inbreeding coefficient (*F*) is the probability that any allele across the autosome is homozygous and identical by descent. The inbreeding coefficient is calculated from the pedigree (*Fped*). However, in families where consanguineous mating has been practiced for several generations, the inbreeding coefficient inferred by the known pedigree may be an underestimation [3]. Alternatively, the inbreeding coefficient may be calculated by the proportion of the genome that is within ROH [4] (*Froh*). Using a stringent threshold of 200 consecutive homozygous SNPs to define regions that are identical by descent with high confidence, we calculated the proportion of the homozygous autosome for each individual in the cohort (sum of all ROH divided by the covered length of the genome). Results are provided in **Table S2**.

**Table S2**. Estimation of inbreeding coefficient

| Individual ID | Parental relatedness | Expected *Fped* | *Froh* | *Froh/Fped* |
| --- | --- | --- | --- | --- |
| Family DR, III-1 | No relation reported |  | 0.011527 |  |
| Family DR, III-2 | No relation reported |  | 0.022501 |  |
| Family DR, III-3 | No relation reported |  | 0.022631 |  |
| Family DR, III-4 | No relation reported |  | 0.020384 |  |
| Family DR, III-5 | No relation reported |  | 0.002334 |  |
| Family DR, IV-1 | No relation reported |  | 0.008245 |  |
| Family DR, V-1 | First cousin once removed | 0.03125 | 0.044727 | 1.43 |
| Family DR, V-2 | First cousin once removed | 0.03125 | 0.053817 | 1.72 |
| Family DR, V-3 | First cousin once removed | 0.03125 | 0.072329 | 2.31 |
| Family DR, V-4 | First cousin once removed | 0.03125 | 0.068647 | 2.20 |
| Family AM-UC, IV-8 | First cousin | 0.0625 | 0.015055 | 0.24 |
| Family AM-UC, IV-9 | No relation reported |  | 0.001005 |  |
| Family AM-UC, IV-16 | First cousin | 0.0625 | 0.011272 | 0.18 |
| Family AM-UC, V-1 | First + Second cousin | 0.078125 | 0.036068 | 0.46 |
| Family AM-UC, V-2 | First + Second cousin | 0.078125 | 0.086535 | 1.11 |
| Family AM-UC, V-3 | First + Second cousin | 0.078125 | 0.087166 | 1.12 |
| Family AM-UC, V-4 | First + Second cousin | 0.078125 | 0.031682 | 0.41 |
| Family AM-UC, V-5 | First + Second cousin | 0.078125 | 0.04799 | 0.61 |
| Family AM-UC, V-6 | First + Second cousin | 0.078125 | 0.016641 | 0.21 |
| Family AJ, IV-1 | First cousin | 0.0625 | 0.099971 | 1.60 |
| Family AJ, IV-2 | First cousin | 0.0625 | 0.085851 | 1.37 |
| Family AJ, IV-3 | First cousin | 0.0625 | 0.067792 | 1.08 |
| Family AJ, IV-4 | First cousin | 0.0625 | 0.052492 | 0.84 |
| Family AM-CD, V-2 | Second cousin | 0.015625 | 0.011285 | 0.72 |
| Family AM-CD, V-4 | Second cousin | 0.015625 | 0.019279 | 1.23 |
| Family AM-CD, V-5 | Second cousin | 0.015625 | 0.003031 | 0.19 |

**Table S2** shows that even when using a stringent threshold for ROH detection (over 200 consecutive homozygous SNPs), high inbreeding coefficients (*Rroh*) are observed in Family-DR. Specifically, in generation III, although no parental relationship was reported, the results indicate a consanguinity ranging between first cousins once removed to second cousins. In generation V, the observed *Rroh* are consistently higher than expected, and indicate that their parents share additional common ancestors than reported. These findings are in line with the report that higher inbreeding coefficients are expected in populations that have preferred consanguineous mating for many generations [3].

***Simulations for CD cases in family DR***

Any two related members from a consanguineous pedigree who share a common ancestor from both parents, are expected to share a proportion of their genome in ROH. For a pair of siblings, this equals *F* X 0.25 (the fraction of the genome for which two siblings are expected to have an identical genotype.)

The two CD cases in Family DR (III-2 and V-1), shared an overlapping ROH spanning 2.6 Mb on chromosome 16, harbouring *NOD2*. To estimate the probability of this finding, we constructed a hypothetical pedigree where III-2 is the offspring of a consanguineous mating, as was indicated by *Froh*. The level of consanguinity was set to first cousins, the closest relationship possible (traditionally) in this family, probably leading to an overestimation of inbreeding.

Gene dropping simulations were undertaken using a custom script in R. The pedigree structure was encoded using the kinship2 library [5]. The chromosome lengths in centiMorgans were obtained from the Phase II HapMap recombination map [6] (hapmap.ncbi.nlm.nih.gov/downloads/recombination/2011-01_phaseII_B37/) dummy marker set was generated with one marker per centiMorgan across the autosome. Each founder was allocated two alleles for each marker labelled with the founder identifier and suffixed _1 or _2. For each non-founder, meioses were simulated with independent assortment performed under a Binomal model (gene dropping with *P* = 0.5) and recombination between parental chromosomes performed under a Binomial model with *P* = (1-exp(-2*d/100))/2 where d is the distance of separation between markers in cM (in this case d = 1, so *P* = 0.01). This process was repeated 10,000 times. Within each iteration, the founder source of both alleles in both individuals of interest at the pre-specified location was examined to check for identical homozygosity.

In approximately 50% of simulations, the two individuals of interest in family DR shared a ROH at least 1 cM long (**Figure S1**). The probability that both affected individuals of interest were identically homozygous for a specific 1 cM region on chromosome 16 (at the approximate position of *NOD2*) was estimated as *P* =0 .0021.


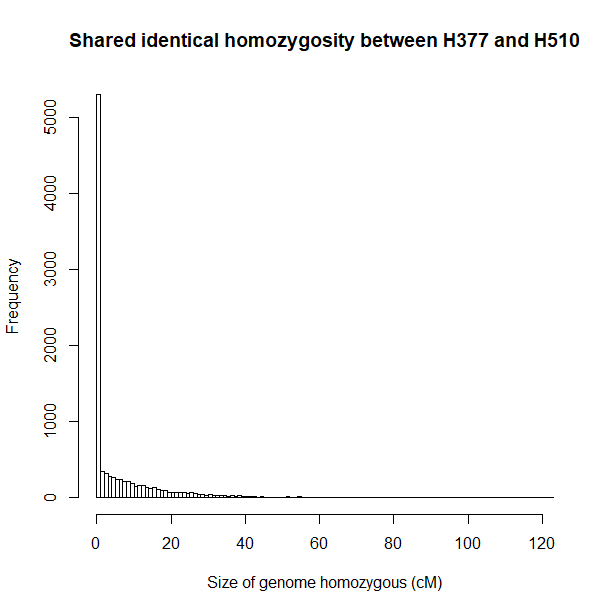


**Figure S1**. Simulations for shared identical and homozygous region between III-2 and V-1.

**Whole exome sequencing**

***Reference population allele frequencies***

Variants were annotated with the Ensembl Variant Effect Predictor tool (VEP) [7] for population frequencies in gnomAD [8]. The Druze and Arab Middle Eastern populations are not represented in gnomAD, possibly leading to incorrect identification of a mutation as uncommon (MAF < 0.05) or rare (MAF < 0.01). Therefore, we included variant frequencies from the Greater Middle East Variome Project (GMEV) [9] (<http://igm.ucsd.edu/gme/>). The GEMV consists of WES derived variants from over 1000 unrelated subjects of Middle Eastern descent.

In three of the families in our cohort with a Middle Eastern ancestry, we found a total of 991 coding variants (15% of coding variants) considered rare in the gnomAD population, but not when allele frequencies from the GMEV are incorporated. For potentially deleterious variants, 316 variants that are are in gnomAD could be excluded (12% of deleterious variants). A detailed description for the potential of the GMEV to exclude common variants otherwise considered rare is provided in **Table S3**.

**Table S3**. Uncommon (MAF < 0.05) and rare (MAF < 0.01) variants in Middle Eastern families with and without incorporation of allele frequencies from the Greater Middle East Variome Project.

| Variants | Druze family | Arab Muslim families | All Middle East families |
| --- | --- | --- | --- |
| Any coding (low impact) | 31478 | 40947 | 47429 |
| Coding + Uncommon Gnomad | 4453 | 8677 | 12411 |
| Coding + Unc Gnomad & GMEV | 4018 | 7924 | 11431 |
| Coding + Rare Gnomad | 2304 | 4385 | 6570 |
| Coding + Rare Gnomad & GMEV | 1925 | 3712 | 5579 |
| Any Damaging | 7847 | 10682 | 12911 |
| Damaging + Uncommon Gnomad | 1661 | 3170 | 4598 |
| Damaging + Unc Gnomad & GMEV | 1546 | 2963 | 4332 |
| Damaging + Rare Gnomad | 938 | 1821 | 2716 |
| Damaging + Rare Gnomad & GMEV | 826 | 1602 | 2400 |

**References**

1. Seelow D, Schuelke M, Hildebrandt F, Nürnberg P. HomozygosityMapper--an interactive approach to homozygosity mapping. Nucleic Acids Res. 2009;37 Web Server issue:W593-9. doi:10.1093/nar/gkp369.

2. Pemberton TJ, Absher D, Feldman MW, Myers RM, Rosenberg NA, Li JZ. Genomic patterns of homozygosity in worldwide human populations. Am J Hum Genet. 2012;91:275–92.

3. Woods CG, Cox J, Springell K, Hampshire DJ, Mohamed MD, McKibbin M, et al. Quantification of homozygosity in consanguineous individuals with autosomal recessive disease. Am J Hum Genet. 2006;78:889–96.

4. Keller MC, Visscher PM, Goddard ME. Quantification of inbreeding due to distant ancestors and its detection using dense single nucleotide polymorphism data. Genetics. 2011;189:237–49.

5. Sinnwell JP, Therneau TM, Schaid DJ. The kinship2 R package for pedigree data. Hum Hered. 2014;78:91–3.

6. International HapMap Consortium TIH, Frazer KA, Ballinger DG, Cox DR, Hinds DA, Stuve LL, et al. A second generation human haplotype map of over 3.1 million SNPs. Nature. 2007;449:851–61.

7. McLaren W, Gil L, Hunt SE, Riat HS, Ritchie GRS, Thormann A, et al. The Ensembl Variant Effect Predictor. Genome Biol. 2016;17:122.

8. Lek M, Karczewski KJ, Minikel E V., Samocha KE, Banks E, Fennell T, et al. Analysis of protein-coding genetic variation in 60,706 humans. Nature. 2016;536:285–91.

9. Scott EM, Halees A, Itan Y, Spencer EG, He Y, Azab MA, et al. Characterization of Greater Middle Eastern genetic variation for enhanced disease gene discovery. Nat Genet. 2016;48:1071–6. doi:10.1038/ng.3592.
