# Supplementary material for: Genetic analysis of four consanguineous multiplex families with inflammatory bowel disease
Source: Gastroenterol Rep (Oxf). 2021 Jul 13;9(6):521–32. doi: 10.1093/gastro/goab007 (PMC8677555; doi:10.1093/gastro/goab007)
Supplement: goab007_Supplementary_Data [file goab007_supplementary_data.zip › Supplementary_Tables_(except_Table_2).docx]

**Supplementary Tables**

**Supplementary Table 1**. IBD associated genes and variants.

**A**. Extensive set of genes associated with IBD

| **Gene name** | **HGNC ID** | **Gene name** | **HGNC ID** | **Gene name** | **HGNC ID** | **Gene name** | **HGNC ID** | **Gene name** | **HGNC ID** | **Gene name** | **HGNC ID** |
| --- | --- | --- | --- | --- | --- | --- | --- | --- | --- | --- | --- |
| ABCB1 | 40 | COG6 | 18621 | GAL3ST2 | 24869 | ITGAL | 6148 | PHACTR2 | 20956 | SPRED1 | 20249 |
| ABCG2 | 74 | COL7A1 | 2214 | GALC | 4115 | ITGAV | 6150 | PHOX2B | 9143 | STARD10 | 10666 |
| ACO2 | 118 | CPEB4 | 21747 | GCKR | 4196 | ITGB2 | 6155 | PIK3R1 | 8979 | STAT1 | 11362 |
| ADA | 186 | CREM | 2352 | GLI1 | 4317 | ITGB8 | 6163 | PLA2G4A | 9035 | STAT3 | 11364 |
| ADAD1 | 30713 | CRP | 2367 | GNA12 | 4380 | ITIH4 | 6169 | PLA2R1 | 9042 | STAT4 | 11365 |
| ADAM17 | 195 | CRTC3 | 26148 | GPR18 | 4472 | ITLN1 | 18259 | PLCG2 | 9066 | STAT5A | 11366 |
| ADAM30 | 208 | CTLA4 | 2505 | GPR183 | 3128 | ITPKA | 6178 | PLCL1 | 9063 | STAT5B | 11367 |
| ADCY3 | 234 | CTSW | 2546 | GPR35 | 4492 | JAK2 | 6192 | pltp | 9093 | STXBP2 | 11445 |
| ADCY7 | 238 | CTSZ | 2547 | GPR65 | 4517 | KIR2DL1 | 6329 | PMPCA | 18667 | TAGAP | 15669 |
| AICDA | 13203 | CUL1 | 2551 | GPX4 | 4556 | KSR1 | 6465 | PNKD | 9153 | TET2 | 25941 |
| AIRE | 360 | CUL2 | 2552 | GRID2IP | 18464 | LACC1 | 26789 | PPP5C | 9322 | TGFBR1 | 11772 |
| ANKZF1 | 25527 | CX3CR1 | 2558 | GRP | 4605 | LCK | 6524 | PRDM1 | 9346 | TGFBR2 | 11773 |
| AQP12B | 6096 | CXCL1 | 4602 | GUCY2C | 4688 | LIG4 | 6601 | PRKCB | 9395 | THADA | 19217 |
| ARIH2 | 690 | CXCL16 | 16642 | HCK | 4840 | LILRB4 | 6608 | PRKCD | 9399 | THEMIS | 21569 |
| ATG16L1 | 21498 | CXCL3 | 4604 | HEATR3 | 26087 | LINC01620 | 16195 | PRKCQ | 9410 | TLR2 | 11848 |
| ATG16L2 | 25464 | CXCL5 | 10642 | HGFAC | 4894 | LITAF | 16841 | PROCR | 9452 | TLR4 | 11850 |
| ATXN2 | 10555 | CXCR5 | 1060 | HIF3A | 15825 | LPP | 6679 | PTGER4 | 9596 | TLR9 | 15633 |
| B3GNT2 | 15629 | CYBA | 2577 | HLA-DQA1 | 4942 | LRBA | 1742 | PTGS2 | 9605 | TMEM17 | 26623 |
| BACH2 | 14078 | CYBB | 2578 | HLA-DQB1 | 4944 | LRRC32 | 4161 | PTK2B | 9612 | TMEM174 | 28187 |
| BANK1 | 18233 | CYTH1 | 9501 | HLA-DRA | 4947 | LRRK2 | 18618 | PTPN2 | 9650 | TMEM180 | 26196 |
| BOK | 1087 | DAP | 2672 | HLA-DRB1 | 4948 | LSP1 | 6707 | PTPN22 | 9652 | TNFAIP3 | 11896 |
| BRD7 | 14310 | DCLRE1C | 17642 | HMHA1 | 17102 | LTBR | 6718 | PTPRK | 9674 | TNFRSF13B | 18153 |
| BRE | 1106 | DENND1B | 28404 | HNF4A | 5024 | MAGI3 | 29647 | PUS10 | 26505 | TNFRSF14 | 11912 |
| BTBD8 | 21019 | DKC1 | 2890 | HNRNPD | 5036 | MAML2 | 16259 | RAG1 | 9831 | TNFRSF18 | 11914 |
| BTK | 1133 | DLD | 2898 | HORMAD2 | 28383 | MAPK1 | 6871 | RAG2 | 9832 | TNFRSF1A | 11916 |
| BTNL2 | 1142 | DLG5 | 2904 | HPS1 | 5163 | MAPKAPK2 | 6887 | RASGRP1 | 9878 | TNFRSF4 | 11918 |
| C11orf30 | 18071 | DMBT1 | 2926 | HPS4 | 15844 | MASP2 | 6902 | RELA | 9955 | TNFRSF6B | 11921 |
| C11orf9 | 1181 | DNMT3A | 2978 | HPS6 | 18817 | MAST3 | 19036 | RET | 9967 | TNFSF11 | 11926 |
| C17orf67 | 27900 | DNMT3B | 2979 | HSPA6 | 5239 | MEFV | 6998 | RFT1 | 30220 | TNFSf15 | 11931 |
| C1orf106 | 25599 | DOCK7 | 19190 | ICOS | 5351 | METTL10 | 33787 | RGS14 | 9996 | TNFSF8 | 11938 |
| C21orf33 | 1273 | DOCK8 | 19191 | ICOSLG | 17087 | MIF | 7097 | RIMBP3 | 29344 | TNIP1 | 16903 |
| C5orf56 | 33838 | DPH5 | 24270 | IFIH1 | 18873 | MMP9 | 7176 | RIPK2 | 10020 | TOM1 | 11982 |
| CALCOCO2 | 29912 | DUSP5 | 3071 | IFNG | 5438 | MST1 | 7380 | RMI2 | 28349 | TRAF3IP2-AS1 | 40005 |
| CAMK2A | 1460 | EGFR | 3236 | IFNG-AS1 | 43910 | MUC1 | 7508 | RNASET2 | 21686 | TRIB1 | 16891 |
| CARD11 | 16393 | ELF1 | 3316 | IFNGR2 | 5440 | MUC19 | 14362 | RNF186 | 25978 | TRIM26 | 12962 |
| CARD9 | 16391 | EPCAM | 11529 | IKBKG | 5961 | MUC2 | 7512 | RORC | 10260 | TRIM31 | 16289 |
| CASP8 | 1509 | EPO | 3415 | IKzF1 | 13176 | MVK | 7530 | RPS6KA2 | 10431 | TRPm2 | 12339 |
| CCDC116 | 26688 | ERAP2 | 29499 | IKZF3 | 13178 | MYO9B | 7609 | RPS6KB1 | 10436 | TSPAN14 | 23303 |
| CCL11 | 10610 | ERGIC1 | 29205 | IL10 | 5962 | NCF1 | 7660 | RTEL1 | 15888 | TTC37 | 23639 |
| CCL13 | 10611 | ETS1 | 3488 | IL10RA | 5964 | NCF2 | 7661 | RTEL1-TNFRSF6B | 44095 | TTC7A | 19750 |
| CCL2 | 10618 | F5 | 3542 | IL10RB | 5965 | NCF4 | 7662 | SATB2 | 21637 | TTYH3 | 22222 |
| CCL20 | 10619 | FADS1 | 3574 | IL17REL | 33808 | NDFIP1 | 17592 | SBSPON | 30362 | TUBD1 | 16811 |
| CCND3 | 1585 | FADS2 | 3575 | IL18RAP | 5989 | NDUFAF1 | 18828 | SCAMP3 | 10565 | TYK2 | 12440 |
| CCR6 | 1607 | FAM171B | 29412 | IL1R2 | 5994 | NFATC1 | 7775 | SDCCAG3 | 10667 | UBE2D1 | 12474 |
| CCRL2 | 1612 | FAM213A | 28651 | IL1RL1 | 5998 | NFKBIA | 7797 | SEPHS2 | 19686 | UBE2L3 | 12488 |
| CD209 | 1641 | FAM92B | 24781 | IL21 | 6005 | NFKBIZ | 29805 | SH2D1A | 10820 | UQCC | 15891 |
| CD226 | 16961 | FAP | 3590 | IL23R | 19100 | NKX2-3 | 7836 | SKAP2 | 15687 | VDR | 12679 |
| CD28 | 1653 | FBLIM1 | 24686 | IL27 | 19157 | NLRC4 | 16412 | SKIV2L | 10898 | WAS | 12731 |
| CD3G | 1675 | FCGR2A | 3616 | IL2RA | 6008 | NLRP12 | 22938 | SLAIN2 | 29282 | XIAP | 592 |
| CD40 | 11919 | FCGR2B | 3618 | IL2RG | 6010 | NLRP2 | 22948 | SLAMF8 | 21391 | YDJC | 27158 |
| CD40LG | 11935 | FCGR3A | 3619 | IL31RA | 18969 | NLRP7 | 22947 | SLC22A23 | 21106 | ZAP70 | 12858 |
| CD6 | 1691 | FERMT1 | 15889 | IL6ST | 6021 | NOD2 | 5331 | SLC22A4 | 10968 | ZBTB38 | 26636 |
| CDC37 | 1735 | FNDC3A | 20296 | IL7R | 6024 | NR1I2 | 7968 | SLC37A4 | 4061 | ZFP36L1 | 1107 |
| CDH1 | 1748 | FOS | 3796 | IL8RA | 6026 | NUPR1 | 29990 | SLC39A11 | 14463 | ZFP91-CNTF | 33441 |
| CDH13 | 1753 | FOSL1 | 13718 | IL8RB | 6027 | NUSAP1 | 18538 | SLC43A3 | 17466 | ZMIZ1 | 16493 |
| CDKAL1 | 21050 | FOSL2 | 3798 | IMPG2 | 18362 | NXPE1 | 28527 | SLCO6A1 | 23613 | ZNF300P1 | 27032 |
| CDSN | 1802 | FOXD1 | 3802 | IRF4 | 6119 | OSMR | 8507 | SMAD3 | 6769 | ZNF329 | 14209 |
| CEBPG | 1837 | FOXP1 | 3823 | IRF6 | 6121 | OTUD3 | 29038 | SMURF1 | 16807 | ZNF365 | 18194 |
| CEP250 | 1859 | FOXP3 | 6106 | IRGM | 29597 | P2RX7 | 8537 | SNX32 | 26423 | ZNF479 | 23258 |
| CEP72 | 25547 | FUT2 | 4013 | ITCH | 13890 | PARK7 | 16369 | SOCS1 | 19383 | ZNF831 | 16167 |
| CIT | 1985 | G6PC3 | 24861 | ITGA4 | 6140 | PDGFB | 8800 | SP140 | 17133 | ZPBP | 15662 |

**B**. Top 20 IBD associated genes

| **Gene name** | **HGNC ID** | **Associated phenotype** | **Gene name** | **HGNC ID** | **Associated phenotype** | **Gene name** | **HGNC ID** | **Associated phenotype** | **Gene name** | **HGNC ID** | **Associated phenotype** |
| --- | --- | --- | --- | --- | --- | --- | --- | --- | --- | --- | --- |
| ATG16L1 | 21498 | CD | FUT2 | 4013 | CD | IL2RA | 6008 | CD | PTPN22 | 9652 | CD |
| CARD9 | 16391 | IBD | IFIH1 | 18873 | UC | ITGAL | 6148 | UC | SMAD3 | 6769 | IBD |
| CD6 | 1691 | CD | IL10 | 5962 | IBD | MST1 | 7380 | IBD | SP140 | 17133 | CD |
| ERAP2 | 29499 | CD | IL18RAP | 5989 | IBD | NOD2 | 5331 | CD | TNFSF8 | 11938 | IBD |
| FCGR2A | 3616 | IBD | IL23R | 19100 | IBD | NXPE1 | 28527 | UC | TYK2 | 12440 | IBD |

**C**. Variants with predicted funtional role in IBD pathogenesis

| **Gene Name** | **Phenotype** | **Direction of Effect** | **SNP** | **Consequence** | **Gene Name** | **Phenotype** | **Direction of Effect** | **SNP** | **Consequence** |
| --- | --- | --- | --- | --- | --- | --- | --- | --- | --- |
| NOD2 | CD | Risk | rs2066847 | fs1007insC | IFIH1 | UC | Risk | rs35667974 | I923V |
| NOD2 | CD | Risk | rs2066844 | R702W | LRRK2 | CD | Risk | rs33995883 | N2081D |
| NOD2 | CD | Risk | rs2066845 | G908R | LRRK2 | CD | Protection | rs7308720 | N551K |
| NOD2 | CD | Risk | rs5743271 | N289S | IL23R | IBD | Protection | rs76418789 | G149R |
| NOD2 | CD | Risk | rs104895444 | V793M | IL23R | IBD | Protection | rs41313262 | V362I |
| NOD2 | CD | Risk | rs104895467 | N852S | IL23R | IBD | Protection | rs11209026 | R381Q |
| NOD2 | CD | Risk | [rs104895447](https://ibd.broadinstitute.org/variant/16-50750842-A-G) | M863V | IRGM | CD | Risk | rs10065172 | c.313C>T |
| NOD2 | CD | Risk | [rs5743277](https://ibd.broadinstitute.org/variant/16-50745929-C-T) | R703C | CARD9 | IBD | Protection | rs141992399 | c.IVS11+ 1G> C |
| NOD2 | CD | Risk | [rs104895431](https://ibd.broadinstitute.org/variant/16-50745114-C-T) | S431L | RNF186 | UC | Risk | rs41264113 | A64T |
| NOD2 | CD | Risk | [rs104895427](https://ibd.broadinstitute.org/variant/16-50744753-C-T) | R311W | RNF186 | UC | Protection | rs36095412 | R179X |
| ATGL16L1 | CD | Risk | rs2241880 | T300A | SMAD3 | IBD | Risk | rs35874463 | I170V |

**Supplementary Table 2**. Genes located within regions of homozygosity.

*Please see separated EXCEL file.*

**Supplementary Table 3**. Summary of whole exome sequencing variants.

|  |  | **Family** | **Average** | **AJ** | **DR all** | **DR Siblings** | **AM-CD** | **AM-UC all** | **AM-UC Siblings** |
| --- | --- | --- | --- | --- | --- | --- | --- | --- | --- |
| **Any variant^a^** | All variants | All | 19524.2 | 19160.5 | 19143.3 | 18546.3 | 19997.8 | 19747.0 | 19594.5 |
|  |  | Uncommon | 1412.4 | 1124.5 | 1343.9 | 1285.8 | 1567.6 | 1494.1 | 1490.2 |
|  |  | Rare | 620.2 | 322.5 | 622.4 | 597.3 | 734.4 | 666.1 | 664.8 |
|  |  | Very rare | 273.1 | 43.8 | 307.1 | 296.3 | 326.4 | 296.9 | 300.5 |
|  | Damaging variants ^b^ | Uncommon | 416.1 | 369.8 | 408.3 | 391.5 | 445.2 | 425.2 | 417.3 |
|  |  | Rare | 221.2 | 130.5 | 224.4 | 217.3 | 247.8 | 236.5 | 238.3 |
|  |  | Very rare | 108.3 | 19.8 | 123.4 | 116.5 | 129.6 | 115.6 | 118.7 |
| **Homozygous variants** | All variants | All | 6749.6 | 6980.8 | 6718.0 | 6670.3 | 6574.2 | 6770.2 | 6860.5 |
|  |  | Uncommon | 39.7 | 42.3 | 41.5 | 61.8 | 24.8 | 43.3 | 58.8 |
|  |  | Rare | 11.8 | 10.0 | 15.4 | 24.3 | 4.2 | 12.6 | 16.5 |
|  |  | Very rare | 5.4 | 0.8 | 7.9 | 12.3 | 1.2 | 6.5 | 9.3 |
|  | Damaging variants ^b^ | Uncommon | 11.1 | 13.3 | 11.4 | 17.5 | 8.2 | 11.4 | 15.3 |
|  |  | Rare | 3.8 | 3.3 | 4.2 | 7.8 | 1.2 | 4.8 | 6.5 |
|  |  | Very rare | 2.1 | 0.5 | 2.0 | 4.3 | 0.4 | 3.2 | 4.8 |

**Abbreviations:** AJ, Ashkenazi Jewish family; DR, Druze family; AM-CD, Arab Muslim family with CD as the major phenotype; AM-UC, Arab Muslim family with UC as the major phenotype.

The table describes the average number of exome-wide (including splice region variants) Reference Sequence variants in in each family. In family AJ, only generation IV (Figure 1D) was considered. In families DR and AM-UC (Figures 1B and 1C), the pedigree structure defines the recent level of consanguinity for the probands and their siblings, and therefore the number of homozygous variants was considered for the entire family and again separately for the probands and their siblings. To avoid confusion, this table does not include variants in the X chromosome, which are hemizygous in males.

^a^ Heterozygous or homozygous variants (each counted once).

^b^ Damaging variants are either frameshift, start, stop or splice donor / acceptor variants, or missense variants predicted to be deleterious by either PolyPhen, SIFT or CADD>20. Uncommon: MAF < 0.05, Rare: MAF < 0.01, Very Rare: MAF < 0.001.

**Supplementary Table 4**. Homozygous damaging variants in IBD associated genes.

|  |  | **All Cases** | **All Controls** | **AJ cases** | **AJ control** | **DR**  **cases** | **DR control** | **AM-CD**  **cases** | **AM-CD control** | **AM-UC**  **cases** | **AM-UC control** | **All CD cases** | **All UC cases** |
| --- | --- | --- | --- | --- | --- | --- | --- | --- | --- | --- | --- | --- | --- |
| **Extensive IBD associated genes**  **(366 genes)** | Any allele frequency | 18.6 | 18.4 | 19.7 | 16.0 | 20.0 | 21.7 | 20.5 | 20.7 | 13.0 | 12.3 | 19.7 | 16.0 |
|  | Uncommon | 0.6 | 0.1 | 0.7 | 0.0 | 0.0 | 0.0 | 1.0 | 0.0 | 1.0 | 0.5 | 0.6 | 0.7 |
|  | Rare | 0.2 | 0.0 | 0.7 | 0.0 | 0.0 | 0.0 | 0.0 | 0.0 | 0.0 | 0.0 | 0.3 | 0.0 |
| **Top genes**  **(20 genes)** | Any allele frequency | 1.6 | 1.7 | 1.7 | 1.0 | 2.3 | 3.0 | 2.0 | 1.7 | 0.0 | 0.0 | 2.0 | 0.7 |
|  | Uncommon | 0.0 | 0.0 | 0.0 | 0.0 | 0.0 | 0.0 | 0.0 | 0.0 | 0.0 | 0.0 | 0.0 | 0.0 |
|  | Rare | 0.0 | 0.0 | 0.0 | 0.0 | 0.0 | 0.0 | 0.0 | 0.0 | 0.0 | 0.0 | 0.0 | 0.0 |

**Abbreviations:** AJ, Ashkenazi Jewish family; DR, Druze family; AM-CD, Arab Muslim family with CD as the major phenotype; AM-UC, Arab Muslim family with UC as the major phenotype.

The table describes the average number of *damaging* and *homozygous* variants per individual in IBD associated genes. To avoid misinterpretation due to pedigree structure, only cases with siblings are included in this analysis, and only unaffected siblings serve as controls.

**Supplementary Table 5**. Homozygous and damaging variants in probands

|  | Variant ID | Chr | Allele | rsID | Gene name ^a^ | Strand | Consequence | Protein position | Amino acids | SIFT | PolyPhen | CADD | gnomAD MAF | Population-specific MAF ^b^ |
| --- | --- | --- | --- | --- | --- | --- | --- | --- | --- | --- | --- | --- | --- | --- |
| AM-CD | 2_167298015_C_T | 2 | T | rs116825611 | SCN7A | -1 | missense | 683 | R/Q | deleterious | Probably damaging | 33 | 0.005 | 0.012 |
|  | 5_38407060_C_T | 5 | T | rs138323038 | EGFLAM | 1 | missense | 320 | T/M | deleterious | benign | 17.71 | 0.014 | 0.029 |
|  | 8_132051970_C_A | 8 | A | rs75246765 | ADCY8 | -1 | missense | 204 | A/S | tolerated | Possibly damaging | 23.4 | 0.014 | 0.012 |
|  | 10_135025208_A_G | 10 | G | rs41283313 | KNDC1 | 1 | missense | 1361 | K/R | deleterious | benign | 19.29 | 0.025 | 0.023 |
|  | 10_134680995_C_T | 10 | T | rs150661681 | TTC40 | -1 | missense | 1543 | A/T | deleterious | Possibly damaging | 23.3 | 0.013 | - |
|  | 10_124345796_C_G | 10 | G | rs143892520 | **DMBT1** | 1 | missense | 560 | D/E | deleterious | unknown | 12.66 | 0.015 | - |
|  | 10_123325158_G_A | 10 | A | rs56226109 | FGFR2 | -1 | missense | 57 | S/L | tolerated | benign | 22.4 | 0.005 | 0.007 |
|  | 10_51225388_A_T | 10 | T | rs200580701 | AGAP8 | -1 | missense | 532 | S/T | tolerated | Possibly damaging | 7.342 | 0.011 | 0.034 |
|  | 12_15800074_G_A | 12 | A | rs76688635 | EPS8 | -1 | missense | 519 | R/C | deleterious | benign | 24.2 | 0.013 | 0.028 |
| AM-UC | 1_248844830_C_G | 1 | G | rs147772378 | OR14I1 | -1 | missense | 259 | G/A | deleterious | benign | 0.003 | 0.001 | 0.007 |
|  | 1_246922356_G_A | 1 | A | rs151266325 | SCCPDH | 1 | missense | 239 | R/Q | tolerated | benign | 20 | 0.001 | 0.003 |
|  | 1_155160698_A_T | 1 | T | rs35819649 | **MUC1** | -1 | missense | 277 | S/T | deleterious | Probably damaging | 23.3 | 0.001 | 0.024 |
|  | 2_20202930_G_A | 2 | A | rs77245812 | MATN3 | -1 | missense | 303 | T/M | deleterious | Probably damaging | 32 | 0.015 | 0.021 |
|  | 6_32020512_T_C | 6 | C | rs17207895 | TNXB | -1 | missense | 3017 | K/R | - | Possibly damaging | 23.3 | 0.024 | 0.032 |
|  | 8_142176436_G_T | 8 | T | - | DENND3 | 1 | missense | 487 | R/S | deleterious | Possibly damaging | 18.83 | - | - |
|  | 8_133899575_G_A | 8 | A | rs2069548 | TG | 1 | missense | 653 | G/D | deleterious | Probably damaging | 24.1 | 0.014 | 0.029 |
|  | 9_131186549_C_G | 9 | G | rs146651928 | CERCAM | 1 | missense, splice region | 187 | Q/E | deleterious | Probably damaging | 22.9 | 0.001 | 0.001 |
|  | 9_127616565_C_T | 9 | T | - | WDR38 | 1 | missense | 51 | R/W | deleterious | Possibly damaging | 28.4 | - | - |
|  | 9_125152621_G_A | 9 | A | rs5794 | PTGS1 | 1 | missense | 481 | V/I | deleterious | benign | 23.3 | 0.007 | 0.015 |
|  | 11_124619648_C_T | 11 | T | rs148389397 | VSIG2 | -1 | missense | 181 | R/H | tolerated | benign | 20.4 | 0.000 | - |
|  | 14_45693239_C_G | 14 | G | rs34402741 | MIS18BP1 | -1 | missense | 851 | E/Q | deleterious | benign | 0.648 | 0.013 | 0.016 |
|  | 14_23845076_G_A | 14 | A | rs148309201 | IL25 | 1 | missense | 174 | R/H | deleterious | Probably damaging | 34 | 0.006 | 0.009 |
|  | 15_67495173_C_T | 15 | T | - | AAGAB | -1 | missense | 312 | D/N | tolerated | Possibly damaging | 25.8 | - | - |
|  | X_140335705_A_T | X | T | rs139422549 | SPANXC | -1 | missense | 80 | L/H | deleterious | Possibly damaging | 10.88 | 0.009 | 0.008 |
| DR | 1_9079349_C_T | 1 | T | rs200486584 | SLC2A7 | -1 | missense | 119 | A/T | deleterious | Probably damaging | 26.3 | 9.34E-05 | 0.003 |
|  | 1_5965455_C_T | 1 | T | rs571655 | NPHP4 | -1 | missense | 618 | E/K | deleterious | benign | 23.3 | 0.010 | 0.031 |
|  | 2_102476165_C_G | 2 | G | rs377671536 | MAP4K4 | 1 | missense | 515 | Q/E | Tolerated (LC) | Possibly damaging | 23.3 | 0.000 | 0.001 |
|  | 2_71211747_C_G | 2 | G | rs374591854 | ANKRD53 | 1 | missense | 304 | H/D | tolerated | Possibly damaging | 23.3 | 0.000 | - |
|  | 2_39519957_C_T | 2 | T | rs148167737 | MAP4K3 | -1 | missense | 410 | A/T | tolerated | benign | 23.9 | 0.001 | - |
|  | 9_107533159_A_G | 9 | G | rs3739740 | NIPSNAP3B | 1 | missense | 154 | K/E | deleterious | Possibly damaging | 27.7 | 0.027 | 0.035 |
|  | 9_107266577_T_C | 9 | C | rs28553883 | OR13F1 | 1 | missense | 12 | F/L | deleterious | Probably damaging | 23.4 | 0.005 | 0.023 |
|  | 10_73544093_C_G | 10 | G | rs74145660 | CDH23 | 1 | missense | 1811 | D/E | tolerated | Probably damaging | 22.4 | 0.014 | 0.015 |
|  | 15_101561275_C_T | 15 | T | rs55798315 | **LRRK1** | 1 | missense | 543 | P/S | deleterious | Probably damaging | 29.8 | 0.004 | 0.017 |
|  | 15_86124616_A_G | 15 | G | rs61731243 | AKAP13 | 1 | missense | 1106 | E/G | Deleterious (LC) | benign | 16.55 | 0.042 | 0.027 |
|  | 15_84611805_C_T | 15 | T | rs61752778 | ADAMTSL3 | 1 | missense | 821 | P/S | tolerated | Probably damaging | 24.4 | 0.016 | 0.009 |
|  | 15_83502031_C_T | 15 | T | rs185529473 | WHAMM | 1 | missense | 725 | R/W | deleterious | Possibly damaging | 29.5 | 4.47E-05 | - |
| AJ | 2_179482089_C_T | 2 | T | rs72677237 | TTN | -1 | missense | 15908 | R/H | - | - | 23.6 | 0.007 | 0.023 |
|  | 2_179442784_C_G | 2 | G | rs72646880 | TTN | -1 | missense | 22820 | A/P | - | - | 22.2 | 0.002 | 0.005 |
|  | 2_113783743_T_C | 2 | C | rs138440701 | IL36B | -1 | missense | 110 | I/V | Deleterious (LC) | benign | 3.083 | 0.013 | 0.022 |
|  | 4_84206004_T_A | 4 | A | rs112033303 | COQ2 | -1 | stop gained | 22 | R/* | - | - | 23 | 0.017 | 0.030 |
|  | 4_70071216_G_A | 4 | A | rs72551397 | UGT2B11 | -1 | missense | 358 | P/S | deleterious | probably_damaging | 23.9 | 0.019 | 0.028 |
|  | 4_39472916_G_A | 4 | A | rs145535775 | LIAS | 1 | missense | 315 | R/H | deleterious | benign | 24.8 | 0.001 | 0.013 |
|  | 5_141035325_G_A | 5 | A | rs144489640 | ARAP3 | -1 | missense, splice region | 1325 | H/Y | tolerated | benign | 13.07 | 0.002 | 0.043 |
|  | 9_133280409_T_C | 9 | C | rs79881769 | AL354898.1 | 1 | missense | 218 | I/T | deleterious | benign | 22.7 | 0.017 | 0.043 |
|  | 9_130890859_C_T | 9 | T | rs148983163 | AL590708.2 | 1 | missense | 25 | S/F | Deleterious (LC) | Possibly damaging | 12.19 | 0.002 | 0.004 |
|  | 9_123785738_G_T | 9 | T | rs34552775 | C5 | -1 | missense | 354 | L/M | deleterious | Possibly damaging | 25 | 0.005 | 0.004 |
|  | 10_50376004_A_G | 10 | G | rs61748312 | C10orf128 | -1 | missense | 16 | V/A | deleterious | benign | 15.43 | 0.039 | 0.029 |
|  | 12_55615229_T_C | 12 | C | rs151030005 | OR10A7 | 1 | missense | 141 | C/R | deleterious | Probably damaging | 17.45 | 0.012 | 0.035 |
|  | 19_17316802_G_A | 19 | A | rs75251420 | **MYO9B** | 1 | missense | 1700 | V/M | deleterious | benign | 23.9 | 0.001 | 0.002 |
|  | 19_14164629_C_T | 19 | T | rs75841596 | PALM3 | -1 | missense | 604 | D/N | deleterious | Possibly damaging | 26.8 | 0.024 | 0.032 |
|  | 22_25750701_C_T | 22 | T | rs138772341 | LRP5L | -1 | missense | 173 | G/R | deleterious | Probably damaging | 26 | 0.0002 | 0.004 |
|  | 22_23154306_C_A | 22 | A | rs780078425 | IGLV3-10 | 1 | missense | 10 | L/I | deleterious | benign | 10.84 | 8.74E-05 | 0.001 |
|  | 22_23040638_T_A | 22 | A | rs185785000 | IGLV2-23 | 1 | missense | 29 | V/E | Deleterious (LC) | Possibly damaging | 23 | 0.012 | 0.037 |
|  | 22_19124865_C_T | 22 | T | rs17743887 | DGCR14 | -1 | missense | 336 | V/M | tolerated | benign | 21.7 | 0.024 | 0.037 |

**Abbreviations** AJ, Ashkenazi Jewish family; DR, Druze family; AM-CD, Arab Muslim family with CD as the major phenotype; AM-UC, Arab Muslim family with UC as the major phenotype; LC, low confidence; gnomAD, Genome Aggregation Database; MAF, minor allele frequency.

^a^ Bold and underlined: gene associated with IBD reported in literature.

^b^ Using MAF in the Greater Middle East Variome (for families AM-CD, AM-UC and DR) or the gnomAD Ashkenazi Jewish population (for family AJ).

**Supplementary Table 6**. Details of prioritized homozygous variants

**A.** Arab Muslim family with CD as the major phenotype (AM-CD)

| Chr | rsID | Gene | Controls | AA change | SIFT | PolyPhen | CADD | gnomAD MAF | GME MAF | Function | Expression | Known association with IBD ^a^ | Homozygous in controls |
| --- | --- | --- | --- | --- | --- | --- | --- | --- | --- | --- | --- | --- | --- |
| 10 | rs56226109 | FGFR2 | missense | S57L | tolerated | benign | 22.4 | 0.0049 | 0.0065 | Fibroblastic growth factor receptor with essential role in regulation of cell proliferation, differentiation, migration and apoptosis | Ubiquitous | miR-595 is upregulated in active IBD and is known to downregulate FGFR2 [76] | 0/3 |
| 10 | rs143892520 | DMBT1 | missense | D560E | deleterious | unknown | 12.66 | 0.015 | - | Secreted glycoprotein scavenger with broad affinity to bacteria and viruses, thought to have a role in innate immunity and mucosal protection | Small intestine and trachea | A deletion polymorphism and SNP in an intronic transcription factor binding site are associated with CD risk [77,78] | 0/3 |

**B.** Arab Muslim family with UC as the major phenotype (AM-UC)

| Chr | rsID | Gene | Controls | AA change | SIFT | PolyPhen | CADD | gnomAD MAF | GME MAF | Function | Expression | Known association with IBD ^a^ | Homozygous in controls |
| --- | --- | --- | --- | --- | --- | --- | --- | --- | --- | --- | --- | --- | --- |
| 8 | - | DENND3 | missense | R487S | deleterious | possibly damaging | 18.83 | - | - | Guanine nucleotide exchange factor activating Rab12, required for autophagy. | Neutrophils | No ^b^ | 1/10 |
| 9 | rs5794 | PTGS1 | missense | V481I | deleterious | benign | 23.3 | 0.0072 | 0.0146 | Involved in the constitutive production of prostaglandins | Smooth muscle cells | No | 3/10 |
| 9 | - | WDR38 | missense | R51W | deleterious | possibly damaging | 28.4 | - | - | Associated with Rab19, unknown role | Nonspecific | No | 3/10 |
| 9 | rs146651928 | CERCAM | missense | Q187E | deleterious | probably damaging | 22.9 | 0.0006 | 0.0005 | Cell adhesion molecule involved in leukocyte transmigration across the blood-brain barrier | Brain | No | 3/10 |
| 15 | - | AAGAB | missense | D312N | tolerated | possibly damaging | 25.8 | - | - | Role in vesicle trafficking, involved in endocytic recycling of growth factor receptors, specifically epidermal GFR | Nonspecific | No | 0/10 |

**C**. Druze family (DR)

| Chr | rsID | Gene | Controls | AA change | SIFT | PolyPhen | CADD | gnomAD MAF | GME MAF | Function | Expression | Known association with IBD ^a^ | Mother (UC) | Uncle (CD) | Uncle (UC) | Homozygous in controls |
| --- | --- | --- | --- | --- | --- | --- | --- | --- | --- | --- | --- | --- | --- | --- | --- | --- |
| 2 | rs148167737 | MAP4K3 | missense | A410T | tolerated | benign | 23.9 | 0.0008 | - | Activates effectors in cell signaling including immune response in T cells. Also involved in response to environmental stress. | Nonspecific | No | X/WT | WT/WT | X/WT | 1/6 |
| 2 | rs377671536 | MAP4K4 | missense | Q515E | tolerated (LC) | possibly damaging | 23.3 | 0.0002 | 0.0005 | Regulates immune response via TNFɑ and IL-6. | Overexpressed in neutrophils upon LPS stimulation | No | X/WT | WT/WT | X/WT | 0/6 |
| 9 | rs3739740 | NIPSNAP3B | missense | K154E | deleterious | possibly damaging | 27.7 | 0.0267 | 0.0347 | Role in vesicle trafficking. | Nonspecific | No | X/WT | X/WT | X/WT | 1/6 |
| 15 | rs185529473 | WHAMM | missense | R725W | deleterious | possibly damaging | 29.5 | 4.47E-05 | - | Actin nucleation-promoting factor. Role in formation of autophagosome through an actin comet tail. | Nonspecific | No | X/WT | X/WT | X/WT | 0/6 |
| 15 | rs61752778 | ADAMTSL3 | missense | P821S | tolerated | probably damaging | 24.4 | 0.0163 | 0.0091 | Glycoprotein in the extracellular matrix, may have a role in cell-matrix interaction or extracellular matrix assembly. | Ubiquitous, nonspecific | No | X/WT | X/WT | X/WT | 0/6 |
| 15 | rs61731243 | AKAP13 | missense | E1106G | deleterious (LC) | benign | 16.55 | 0.0415 | 0.0267 | Scaffold protein with a role in assembling signaling complexes down-stream of several G-protein coupled receptors, including Rho pathway. | Ubiquitous, highly expressed in innate immune cells | No | X/WT | X/WT | X/WT | 0/6 |
| 15 | rs55798315 | LRRK1 | missense | P543S | deleterious | probably damaging | 29.8 | 0.0044 | 0.0171 | GTPase activity, participates in regulation of autophagy | Dendritic cells | Known CD GWAS locus in this gene [62] | X/X | WT/WT | WT/WT | 1/6 |

**D**. Ashkenazi Jewish family (AJ)

| Chr | rsID | Gene | Controls | AA change | SIFT | PolyPhen | CADD | gnomAD MAF | gnomAD AJ MAF | Function | Expression | Known association with IBD ^a^ | IV-2 | IV-3 | Homozygous in controls ^c^ |
| --- | --- | --- | --- | --- | --- | --- | --- | --- | --- | --- | --- | --- | --- | --- | --- |
| 4 | rs112033303 | COQ2 | stop gained | R22X | - | - | 23 | 0.0166 | 0.0304 | Enzyme that catalyzes the final steps in the biosynthesis of CoQ (ubiquinone) | Ubiquitous | No | X/X | X/X | 0–1/3 |
| 2 | rs138440701 | IL36B | missense | I110T | deleterious (LC) | benign | 3.083 | 0.013 | 0.0222 | Member of the IL1 family, signals through the IL36 receptor which in turn activates NF-kappa-B and MAPK signaling pathways | Immune cells and several tissues, keratinocytes | No. IL36A and IL36G are overexpressed in inflamed mucosal tissue from IBD patients [79] | X/X | X/WT | 0–1/3 |
| 9 | rs34552775 | C5 | missense | L354M | deleterious | possibly damaging | 25 | 0.0054 | 0.0044 | Component of the complement system; C5a is a potent spasmodic and chemoattractant, and C5b is a subunit of the membrane attack complex | Hepatocytes | Complement activation is increased in IBD tissue, but no association with variants in complement genes | X/WT | X/X | 0–1/3 |
| 19 | rs75841596 | PALM3 | missense | D604N | deleterious | possibly damaging | 26.8 | 0.0245 | 0.0319 | ATP-binding protein, may act as adaptor in TLR signaling | Nonspecific | No | X/X | WT/WT | 0/3 |
| 19 | rs75251420 | MYO9B | missense | V1700M | deleterious | benign | 23.9 | 0.0014 | 0.0024 | Unconventional myosin serving in intracellular movement. Also acts as a GTPase for RHOA, role in cell migration and tight junction regulation. | Immune cells, especially monocytes and neutrophils, specifically intestinal monocytes | Several polymorphisms associated with CD and UC risk [80] | X/X | WT/WT | 0/3 |

**Abbreviations**: Chr: chromosome; AA: amino acid; gnomAD, Genome Aggregation Database; GME: Greater Middle East; MAF: minor allele frequency (missing MAF – non-existent in dataset); X and WT refer to the genotype where X is the minor allele and WT the common allele.

Family-specific variants derived from whole exome sequencing. Variants were prioritized if they satisfied all of the following: homozygous, predicted to be damaging, allele frequency < 0.05, segregated in the affected cases and could not be excluded based on an unrelated specific function or gene expression.

^a^ Known association with IBD, "No": Gene is not a member of the “extensive” list of 366 IBD associated genes, PubMed search for key words "Crohn", "colitis" and "inflammation" together with the gene or protein name did not identify any reports of a role in IBD, and query for the specific variant and gene in the IBD Exome Browser does not indicate association.

^b^ The specific variant does not exist in IBD exome browser, the gene has four risk variants with *P* < 0.01.

^c^ In three unaffected siblings. Estimation for homozygosity for the deceased siblings was based on WES from their descendants.
